# Linalool and 1,8‐Cineole as Constitutive Disease‐Resistant Factors of Norway Spruce Against Necrotrophic Pathogen *Heterobasidion Parviporum*

**DOI:** 10.1111/pce.15280

**Published:** 2024-11-13

**Authors:** Kai Wang, Wenzi Ren, Liang Hong, Qingao Wang, Rajendra Ghimire, Matti Haapanen, Minna Kivimäenpää, Pengfei Wu, Xiangqing Ma, Fred O. Asiegbu

**Affiliations:** ^1^ College of Forestry Fujian Agriculture and Forestry University Fuzhou China; ^2^ Department of Forest Sciences University of Helsinki Helsinki Finland; ^3^ Department of Environmental and Biological Sciences, Kuopio Campus University of Eastern Finland Kuopio Finland; ^4^ Natural Resources Institute Finland (LUKE) Helsinki Finland; ^5^ Natural Resources Institute Finland (LUKE) Suonenjoki Finland

**Keywords:** constitutive disease resistance, necrotrophic pathogen, terpene, transcriptome

## Abstract

Norway spruce is an important coniferous species in boreal forests. Root and stem rot diseases caused by the necrotrophic pathogen *Heterobasidion parviporum* threaten the wood production of Norway spruce which necessitates the search for durable control and management strategies. Breeding for resistant traits is considered a viable long‐term strategy. However, identification of potential resistant traits and markers remains a major challenge. In this study, short‐term disease resistance screening was conducted using 218 Norway spruce clones from 17 families. Disease resistance was evaluated based on the size of necrosis lesion length following infection with the pathogen. A subset of needles/branches from clones with small (partial resistant) or large (susceptible) lesions were used for terpene analysis and transcriptomic profiling. The results revealed that the content of monoterpene linalool and 1,8‐cineole and their respective encoded genes were significantly more abundant and highly expressed in the partial resistant group. Furthermore, linalool and 1,8‐cineole were demonstrated to have inhibitory effect on the growth of the pathogen *H. parviporum*, with morphological distortion of the hyphae. RNAseq analysis revealed that transcript of pathogen genes involved in the regulation of carbohydrate metabolism and stress responses were significantly decreased in presence of the terpenes. The results suggest the relevance of monoterpenes together with jasmonic acid precursor and some genes involved in phenylpropanoid biosynthesis, as constitutive tolerance factors for Norway spruce tolerance against necrotrophic pathogen. The high level of necrosis related cell death gene expression might be factors critical for host susceptibility and disease development.

## Summary statement

In this study, disease resistance screening was conducted using 218 Norway spruce clones from 17 families. We revealed that the relevance of monoterpenes together with jasmonic acid precursor and some genes involved in phenylpropanoid biosynthesis, as constitutive tolerance factors for Norway spruce tolerance against necrotrophic pathogen.

## 1 Introduction

Norway spruce (*Picea abies* (L.) Karst), with pronounced economic and ecological values, is a major coniferous species in boreal forests in Europe. Due to the shallow root system, spruce trees are susceptible to abiotic disturbance factors such as drought, heat, wind‐throw and rock‐fall (Caudullo, Tinner, and de Rigo 2016). More severely, spruce forests are affected by biotic stresses, such as root and stem rot disease, bark beetles, etc. The species complex *Heterobasidion annosum*, which exists worldwide, causes root and stem rot disease in conifer trees in Northern Europe (Asiegbu, Adomas, and Stenlid 2005; Kovalchuk et al. 2013). The species complex consists of five species, among which the fungus *Heterobasidion parviporum* (Hp) prefers to attack Norway spruce. *Heterobasidion* spp. infect Norway spruce via trunk wounding or freshly cut stumps and transmit from tree to tree through root‐to‐root contact. This pathogen causes decay within heartwood that extends to sapwood on adult trees as well as kills young trees (Asiegbu, Adomas, and Stenlid 2005). Since *H. parviporum* is a necrotrophic pathogen, with a dual nutritional survival strategy by feeding on living and dead tissues, the fungus is difficult to eradicate after it occurs in a forest. Multiple approaches have been used to control root rots, such as stump removal coupled with the stump treatment with urea or a biological fungal agent *Phlebiopsis gigantea* (Garbelotto and Gonthier 2013; Kärhä et al. 2018). Nevertheless, the external treatment or control approaches is expensive and time‐consuming. Therefore, for the long‐term purpose, aiming for innate in forest trees to avoid the initial infection provides a durable solution to manage root rot diseases.

Plant resistance to biotrophic pathogens usually depends on major resistance genes, which result in a hypersensitive response and cell death to limit the spread of biotrophic pathogens (Chisholm et al. 2006; Dangl and Jones 2001). Resistance to necrotrophic pathogens, however, is a much more complex trait and mostly unclear. Preformed resistance is a type of constitutive resistance that exists before encounter with any pathogen. As an important approach of preformed resistance, coniferous trees produce terpenes that usually act as a defensive barrier to resist pathogens (Bonello et al. 2006; Keeling and Bohlmann 2006; Mahizan et al. 2019). Oleoresin terpenoids for example, monoterpenes, sesquiterpenes and diterpenes, together with phenolics for example, stilbenes, flavonoids and lignin are prominent secondary metabolites associated with defense and resistance to pathogens in conifer trees (Kovalchuk et al. 2013; Bullington et al. 2018; Donoso et al. 2015). Higher content of terpenes can be induced in Norway spruce by fungi priming, which has been shown to induce resistance against bark beetle (Mageroy et al. 2020; Novak et al. 2014). Different compositions of terpene compounds have been reported in constitutive and induced terpenoid resin for resistance outcome (Martin et al. 2002; Axelsson et al. 2020). Factors such as physical wounding and insect or fungal attacks can induce the production of stilbenes, flavonoids and lignin in many conifer species, and these compounds have impacts on defense through increasing mechanical strength and anti‐pathogen/insect activities (Kolosova and Bohlmann 2012). Concerning the resistance to *H. parviporum* in Norway spruce, it has been shown that genotypes with multiple alleles of gene encoding for leucoanthocyanidin reductase were less susceptible (Nemesio‐Gorriz et al. 2016). Genes involved in the lignin and flavonoid biosynthesis, as well as certain terpene and phenolic compounds may determine resistance to root and stem rots (Kovalchuk et al. 2019; Liu et al. 2022).

Considering the importance of preformed resistance in limiting pathogen invasion at early stage, selecting this type of trait is vital in conifer breeding programs. Candidate genetic and chemical markers will assist in predicting resistant genotypes and consequently enhancing resistance to root and stem rot diseases. Breeding for natural genetic resistance is based on a few basic steps, such as short‐term resistance screening using artificial inoculation and field tests to evaluate the resistance durability (Sniezko et al. 2014). Although breeding trees showing resistance to pests or diseases needs sustained supports from many aspects, successes have been achieved in a few well‐planned cases, such as white pine blister rust resistance in white pines and white pine weevil resistance in Sitka spruce (Sniezko and Koch 2017; Woodcock, Marzano, and Quine 2019). Furthermore, omics approaches including transcriptomic, metagenomic and metabolomic profiling have facilitated breeding programs to identify genetic factors (Naidoo et al. 2019).

Our previous studies with small group of clonal materials (70 clones) have demonstrated that Norway spruce resistance to *Heterobasidion* involves multiple distinct signaling pathways and regulatory factors (Liu et al. 2021, 2022). Higher contents of monoterpenes and sesquiterpenes, as well as the upregulation of flavonoid biosynthesis genes and peroxidase genes correlated with resistant trait (Liu et al. 2021, 2022). Yet, resistance screening from a larger clonal population is necessary to confirm and identify additional resistant markers. Moreover, potential factors contributing to constitutive resistance and susceptibility merits further detailed investigation. The experimental evidence on direct or indirect inhibitory effect to Hp by host metabolites has not yet been fully studied.

In this study, we screened and identified partially resistant and highly susceptible Norway spruce clones by large‐scale phenotyping of the necrosis development traits from 218 clones. Combined with terpene metabolite abundance, terpene antifungal effects, transcriptomic and WGCNA analysis, the constitutive resistant genes and metabolites that might contribute to Norway spruce partial resistance to necrotrophic pathogen Hp were unraveled. Notably, both the content and gene expression level of monoterpene linalool and 1,8‐cineole were significantly more abundant in partial resistant group. Direct antifungal effects of 1,8‐cineole, linalool and mixture of the two chemical compounds were validated against Hp. Screening for constitutive resistance aids the discovery of gene candidates, which could be applied as markers for genomic selection in resistance tree breeding.

## 2 Material and Methods

### 2.1 Norway Spruce Cultivation and Hp Infection

The plant materials for this study were provided by the Finnish spruce breeding program, which created 17 full‐sib families through controlled crosses. Each full‐sib family consisted of 7 to 15 trees, totaling 218 trees, which the Natural Resources Institute Finland (Luke) propagated as cuttings. Eight cuttings were produced per each clone. The final study material was provided as 3‐year‐old rooted cuttings (altogether 2180 cuttings) in March 2020. The cuttings were transplanted into 15 × 15 × 15 cm pots with peat substrate Kekkilä FPM 420 (Kekkilä Professional, Finland) in greenhouse. The setting of greenhouse was 23/18°C 16/8 h, with curtain shed with sunlight strength > 200 W/m^2^. Plants were kept in greenhouse for 2 weeks before RNA sample collection and Hp infection. Five clonal plants were used for Hp infection and three plants for control (inoculated with media). For Hp infected trees, 42–44 clones (five plants) were randomly selected for locating on one table. The cuttings of each clone were placed randomly on the table. Infection and control groups were kept separate to avoid cross infection. Tree height of all individuals was measured before and 4 months after the infection.

*H. parviporum* heterokaryotic isolates (04009, Figure S1), which was the isolate with strongest virulence among our tested isolates, were cultivated on malt extract sawdust agar plates (MEA‐S; 2% malt extract, 2% Norway spruce sawdust and 1.5% agar) at 20°C for 2 weeks before infection. The hyphae reached the outer edge of plate (9 cm in diameter) after 2‐week cultivation. Young hyphae (close to plate edge) were used as the inoculum material. Specifically, we used 5 mm sterilized puncher to harvest inoculum plugs from the agar media. The distances between the punched sites and original inoculated site were the same, ensuring all the inoculation material have the same vigor.

**Figure 1:**
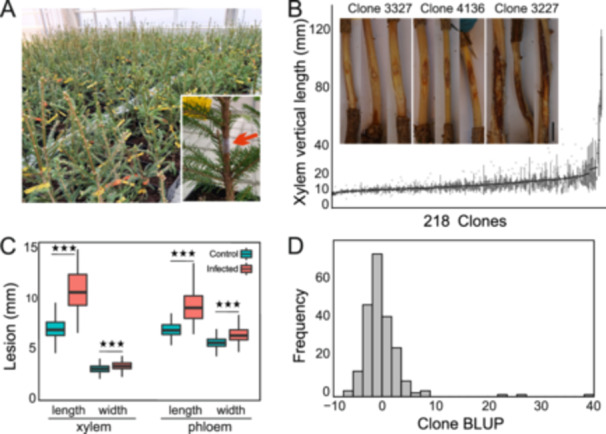
Summary of Norway spruce tolerance by screening lesions of *Heterobasidion parviporum* infection. (A) Photos of Norway spruce clones used in this study. Red arrow indicated the site of infection. (B) Summary of lesion xylem vertical length among 218 clones (*n* = 5) infected with *H*. *parviporum* and lesion photos of representative trees. Lesion data was plotted with ggplot2 in R, middle line represented the median, the box upper and lower ends were 75% and 25%. (C) Comparison of lesion length from *H*. *parviporum* infected and control clones. Four indicators: xylem vertical, xylem horizontal, phloem vertical and phloem horizontal length were measured. (D) Clone best linear unbiased prediction (BLUP) values were calculated with linear model with random effects for variance components using lme4 package in R.

The infection hole on tree stem was made with a 70% ethanol‐sterilized puncher (5 mm in diameter) to reach the xylem surface, with phloem and cambium tissues removed. The distance of infection sites from the stem base was about 5 cm. The MEA‐S agar plugs with Hp hyphae were utilized for infection. Trees treated with the sterilized puncher and pure MEA‐S plugs were used as control. The infection sites were sealed with Parafilm M (Heathrow Scientific, USA) immediately after infection. The infection period was 4 months (Figure 1A). The experimental flow was depicted in Figure S2.

### 2.2 Lesion Measurement, Heritability and Genetic Correlations

The above‐ground parts of Norway spruce clones were harvested after 4 months post infection. Phloem and cambium tissues around the infection site were carefully removed with a knife. The vertical and horizontal length of lesion area in both xylem and phloem were measured in Fiji ImageJ 1.52 (Schneider, Rasband, and Eliceiri 2012) from 2180 lesion photos. The difference in lesion sizes between wounded and infected cuttings was estimated by a paired t‐test of clonal replicate means calculated by plyr 1.8.6 (Wickham 2011). Lesion size was plotted using ggplot2 in R 4.2.3. Best linear unbiased prediction (BLUP) of length size was calculated for each clone using lme4 package (Bates et al. 2015) in R 4.2.3. Variance components include clonal, clonal family, block and pathogen batch. Based on the BLUP values and lesion sizes, we selected five partial resistant (PR) and three susceptible (S) clones as representatives for terpenes quantification and RNA sequencing.

A standard univariate mixed model was used to divide the variation in lesion length and height difference into causal components *y* = Xb + Zu + *e*, where *b* is a vector of fixed effects (the general mean), *u* is a vector of random additive genetic effects among clones within full‐sib families (distributed with the expected value *E*(*u*) = 0 and Var(*u*) = Aσ^2^_A_) and e is a vector of random residuals (*E*(*e*) = 0 and Var(*e*) = *σ* ^2^_*E*_). *σ* ^2^_*A*_ is the additive genetic variance and A is the numerator additive relationship matrix. *X* and *Z* are incidence matrices. Narrow‐sense heritability on a clone‐mean basis was calculated as follows: *h* ^2^ = *σ* ^2^_*A*_/(*σ* ^2^_*A*_ + *σ* ^2^_*E*_/*n*_*H*_), where *n*_*H*_ is the harmonic mean of the ramets per clone (in this case, *n*_*H*_ = 4.18).

Genetic correlations between the four dimensions of lesion trait (lesion height in phloem, lesion height in xylem, lesion area in phloem, lesion area in xylem) were estimated according to the formula *r*_*G*(*x*,*y*)_ = *σ* ^2^_*A*(*x*,*y*)_ (*σ* ^2^_*A*(*x*)_ *σ* ^2^_*A*(*y*)_)^−2^, where *σ* ^2^_*A*(*x*,*y*)_ is the additive genetic covariance between two traits (*x*, *y*) and *σ* ^2^_*A*(*x*)_ and *σ* ^2^_*A*(*y*)_ denote the additive genetic variance components of each of the traits. The variance and covariance components were estimated based on a multivariate extension of the mixed model analysis (Isik, Holland, and Maltecca 2017). The (phenotypic) correlations between traits on a clone‐mean basis (*r*_*P*(*x*,*y*)_) were calculated using estimated variance components from the same analysis, *r*_*P*(*x*,*y*)_ = (*σ* ^2^_*A*(*x*,*y*)_ + *σ* ^2^_*E*(*x*,*y*)_/*n*_*H*_)/((*σ* ^2^_*A*(*x*)_ + *σ* ^2^_*E*(*x*)_/*n*_*H*_) (*σ* ^2^_*A*(*y*)_ + *σ* ^2^_*E*(*y*)_/*n*_*H*_))^−2^, where *σ* ^2^_*E*(*x*,*y*),_ *σ* ^2^_*E*(*y*),_ σ^2^_*E*(*y*)_ denote the environmental covariances and variances of the two traits (here referred to as *x* and *y*). All the analyses were performed using the software package ASReml (Gilmour et al. 2015). The height increase during infection period (4 months) was recorded as: *h*_increase_ = *h*_after_ − *h*_before_. The correlation between tree height increases and lesion length was calculated as mentioned above.

### 2.3 Terpenes Determination and Hp Inhibition Test

Monoterpenes and sesquiterpenes from 8 Hp infected clones (23 individual plants for the PR group and 8 for the S group) were quantified using a previously described method (Liu et al. 2022; Kainulainen et al. 1992). Norway spruce needles and young stem adjacent to the infection cite were collected before pathogen Hp infection. Frozen sample was grinded in the liquid nitrogen and approximately 100 mg powder was used for extraction in 2 mL of n‐hexane at room temperature for 2 h and washed twice with 2 mL n‐hexane. 1‐chloro‐octane (70.1 g) was applied as an internal standard. Chloro‐octane was used because it is similarly soluble in n‐hexane and eluted similarly to monoterpenes and sesquiterpenes on the GC column, is not present in the actual samples, and does not co‐elute with monoterpenes and sesquiterpenes (Kainulainen et al. 1992). Details of gas chromatography–mass spectrometry has been described earlier (Liu et al. 2022). The mass spectra, retention time and authentic standard compounds were used to identify and quantify monoterpene and sesquiterpene compounds. For each detected terpene, the concentration comparison between PR and S groups were assessed with two‐tailed unpaired *t*‐test in R 4.2.3. All the individuals in each group were considered in *t*‐test. Furthermore, in this study, needle tissues were sampled primarily to identify constitutive chemical or molecular markers in clones found to be either susceptible or resistant. By sampling the needles before pathogen inoculation, we avoided destructive stem inoculation which often poses a technical and logistical challenge and may be impractical for large‐scale screening of novel biomarkers for durable resistance.

For Hp inhibition test, 1,8‐cineole (TCI, Shanghai, C0542) and linalool (Aladdin, Shanghai, L106905) solution was firstly emulsified by adding 2% Tween‐20. The emulsified 1,8‐cineole was diluted into 1:2, 1:4, 1:8, 1:16, 1:32, 1:64 and 1:128 stock. Cooling autoclaved potato dextrose agar (PDA) (about 46°C) was mixed with linalool and 1,8‐cineole stocks at 9:1 ratio and was solidified on Petri dish. The mixture of media and MQ water was applied as control. After solidification, media were covered with sterile cellophane to facilitate hyphae collection afterwards. Hp culture was firstly activated on PDA for a week and were then transferred into the middle of PDA plate containing 1,8‐cineole or linalool or mixture using puncher (5 mm in diameter). Plates were kept at 22°C and the growth of Hp was recorded daily.

### 2.4 Total RNA Extraction and RNAseq Reads Mapping

Plant material was the needles that were located on similar position (1/2 of total height) of 3‐year‐old Norway spruce clones. Needles with the branch were collected in 2‐ml Eppendorf tubes. Hp mycelium inhibition test was conducted on PDA media pre‐covered with sterile cellophane. Both needle and mycelium samples were immediately frozen in liquid nitrogen and kept in −80°C until used. Frozen samples were milled into powder with Mixer Mill MM400 (Retsch technology, Haan, Germany) in 2 mL Eppendorf tube with 5 mm sterile steel ball (60 s, 22 Hz). Total RNA extraction followed previous protocol (Chang, Puryear, and Cairney 1993; Wang et al. 2021). Briefly, Plant tissues were milled for 60 s at 22 Hz with 5 mm sterile steel ball in a Mixer Mill MM400 (Retsch technology, Haan, Germany) within 2 mL Eppendorf tube. Grounded sample was transferred to a sterile 2 mL Eppendorf tube with 900 µL extraction buffer (65°C) and 9 µl DTT (1 mol/L). The tubes were vortexed and incubated at 65°C for 15 min. 900 µL of chloroform: isoamyl alcohol (24:1) were added, followed by mixing and centrifugating at 10 000 g for 10 min at room temperature. The upper phase was transferred to a new 2 mL Eppendorf tube and then equal volumes of chloroform: isoamyl alcohol (24:1) were added for repeating the above step. 1/4 volume of 10 M LiCl (42.4 g/mol) was added to precipitate RNA. Samples were mixed well and kept at 4°C overnight. The tube was centrifuged at 10 000 g at 4°C for 30 min, and supernatant was pipetted out and the pellet was washed by adding 100 µL cold 70% ethanol. The centrifugation was repeated for 5 min. The supernatant was pipetted out and the pellet was dried in sterile hood. The pellet was re‐suspended in 20 µL nuclease‐free water. RNA was quantified by NanoDrop 2000c (Thermo Fisher Scientific, USA) and was checked with Agilent 2100 bioanalyzer (Agilent Technologies, Germany).

Equal amount of high‐quality RNA from 4 to 5 replicates of each clone were gathered into one clone sample. RNA (1 µg) samples were shipped to Novagene (UK) for RNA sequencing. After raw reads quality check with FastQC v0.11.8 and MultiQC v1.8, ribosomal RNAs were filtered with SortMeRNA v4.2.0 (Kopylova, Noé, and Touzet 2012). Adapter sequences and low‐quality reads (Q < 20 in 5‐base sliding windows) were trimmed with trimmomatic v0.39 (50 bp minimum length) (Bolger, Lohse, and Usadel 2014). The yield reads were qualified again with FastQC and MultiQC. Processed reads were mapped against the genome of *P. abies* or *H. parviporum* (assembly ASM299478v1, PDUQ00000000) with STAR v.2.7.2 (Dobin et al. 2013; Kopylova, Noé, and Touzet 2012), and the genome was downloaded from ConGenIE database (Nystedt et al. 2013). Uniquely mapped reads were checked by htseq‐count script with HTSeq v.0.15.3 (Anders, Pyl, and Huber 2015), which produced raw count tables.

### 2.5 RNAseq, WGCNA and Statistical Analysis

Combined count table was transformed with variance stabilizing transformation (vst) and were normalized for library size and RNA composition effect (Love, Huber, and Anders 2014). Transformed and normalized counts were used for Principal component analysis (PCA) (Love, Huber, and Anders 2014). Differential expressed genes (DEGs) were produced with DESeq. 2 (Love, Huber, and Anders 2014) with adjusted P‐value 0.05 and fold change log2 > 1 (Wang, Wen, and Asiegbu 2022). DEG heatmap (scaled by row) and dendrogram were produced with package gplots heatmap.2 (Warnes et al. 2024) using DESeq. 2 normalized counts in R. Norway spruce gene annotation was applied from genome annotation (Nystedt et al. 2013) and manual check. GO enrichment of DEGs was carried out with package clusterProfiler 4.0 (Wu et al. 2021) with *p*‐value 0.01 and p adjust BH method. Gene‐list Enrichment tool in KOBAS 3.0 (Bu et al. 2021) was applied for KEGG pathway enrichment of DEGs, with Fisher's exact test and FDR (Benjamini‐Hochberg adjusted) < 0.05.

R package WGCNA (1.72‐5) was applied for co‐expression analysis of T and S groups. We combined count tables of T and S groups from both current and previous studies (Langfelder and Horvath 2008). VST transformation was performed after the removal of lowly expressed transcripts with count < 1 in all libraries and count < 100 in at least eight libraries. Gene modules were identified with 1‐step network construction function blockwiseModules with proper soft‐threshold power (power = 6), 100 minimum module and 0.25 merging threshold function. Hub genes in the keymodule were identified using cut‐offs of eigengene‐based connectivity (KME) ≥ 0.8 and gene Trait Significance ≥ 0.2.

## 3 Results

### 3.1 Partial Host Resistance to Necrotrophic Pathogen Heterobasidion Is a Quantitative Trait

To better quantify the lesion development, we measured vertical and horizontal length of lesion area in both xylem and phloem (Figure 1B). The clonal lesions caused by Hp infection are significantly larger than those of control (wounding treated) clones, based on all of four lesion indicators (Figure 1C). This indicated that our Norway spruce *Heterobasidion* infection system was successful and *H. parviporum* isolate 04009 was capable to cause considerable damage to young Norway spruce within 3 months. High correlations among the four lesion indicators were observed (Table 1), suggesting that the lesion development in vertical and horizontal directions in both xylem and phloem is consistent. The vertical length of xylem lesion area was applied as the main indicator to represent the *H. parviporum* development in the following analysis.

**Table 1:** Genetic and phenotypic correlations among four measurement of *Heterobasidion parviporum* infected necrotic lesion, as well as lesions and height increase. Phenotypic correlation were calculated on a clone‐mean basis.

Trait1	Trait2	Genetic correlation		Phenotypic correlation	
		rG	SE (rG)	rP	SE (rP)
Xylem horizontal	Xylem vertical	0.62	0.06	0.59	0.05
Phloem horizontal	Xylem horizontal	0.81	0.04	0.74	0.04
Phloem horizontal	Xylem vertical	0.75	0.05	0.69	0.05
Phloem vertical	Xylem horizontal	0.63	0.06	0.58	0.05
Phloem vertical	Xylem vertical	0.99	0.01	0.96	0.01
Phloem vertical	Phloem horizontal	0.81	0.04	0.74	0.04
Xylem horizontal	Height increase	−0.25	0.31	0.03	0.08
Xylem vertical	Height increase	−0.24	0.32	−0.03	0.08
Phloem horizontal	Height increase	−0.30	0.32	0.00	0.08
Phloem vertical	Height increase	−0.29	0.32	−0.04	0.08

Abbreviations: rG, genetic correlation; rP, phenotypic correlation; SE, standard error.

The length of the xylem lesion ranged from 6.7 mm to 89.0 mm (Figure 1B). Clone‐mean heritability was 0.862 (±0.018) (Table 2). The histogram of BLUP values for xylem vertical length showed near normal distribution (Figure 1D). A strikingly large lesion length was observed for some susceptible clones (Figure 1B). Representative lesion images for partial resistant (clone 3327), medium susceptible (clone 4136) and susceptible clonal group (clone 3227) were shown in Figure 1B. We observed a considerable variation in lesion size across clones representing the same full‐sib family, indicating a low degree of family‐specificity of Hp partial resistance. Despite this, we found some interesting performance differences on Hp resistance among the 17 families. For instance, Family 39 clones are significantly more resistant than clones from Family 27 (Figure S3). We also measured the tree growth during the infection period and correlated the increased height with lesion length. There was no significant correlation between lesion development and growth.

**Table 2:** Clone‐mean heritability of *Heterobasidion parviporum* infected trees.

Lesion	Estimate	Standard error
Xylem vertical	0.862	0.018
Xylem horizontal	0.827	0.022
Phloem vertical	0.814	0.025
Phloem horizontal	0.725	0.036

### 3.2 Constitutive Differential Expressed Genes From Partial Resistant and Susceptible Clones

**Figure 2:**
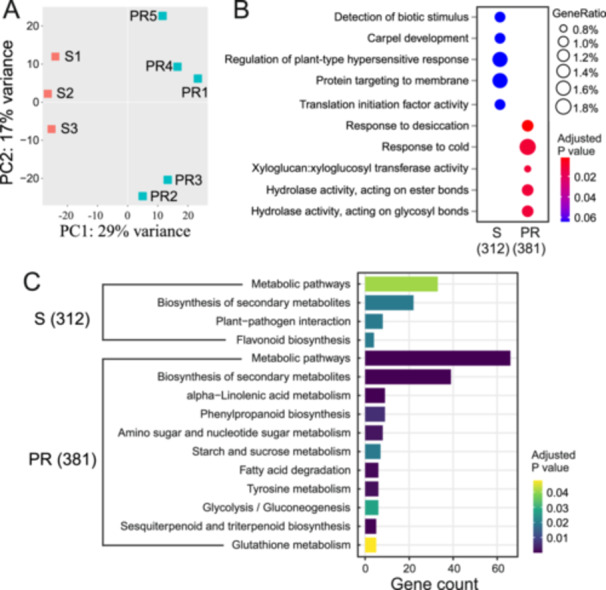
RNAseq profiling of samples of partial resistant and susceptible groups. (A) PCA plot of total uniquely mapped reads of samples from tolerant and susceptible groups. (B) GO enrichment of differential expressed genes of tolerant and susceptible groups. Package clusterProfiler 4.0 was used with *p* value 0.01 and p adjust BH method. (C) KEGG pathway enrichment of differential expressed genes of tolerant and susceptible groups. Gene‐list Enrichment tool in KOBAS 3.0 was applied, with Fisher's exact test and FDR (Benjamini‐Hochberg adjusted) < 0.05. PR, partial resistant group; S, susceptible group. [Color figure can be viewed at wileyonlinelibrary.com]

To reveal the mechanisms of constitutive disease resistance, we assigned the clones with the smallest and largest lesions to two groups, partial resistant (PR) and susceptible (S), respectively. RNAseq analysis was conducted with DEseq. 2 (Love, Huber, and Anders 2014) to compare the constitutive transcriptomic characters for the clones of the PR and S groups. The PR and S groups were well clustered and separated by the first principal component axis which explained 29% of the variance (Figure 2A). GO enrichment suggested that the regulation of plant‐type hypersensitive response was significantly enriched in the S group (Figure 2B). Additionally, KEGG pathway enrichment analysis implied that plant‐pathogen interaction and flavonoid biosynthesis pathways were enriched in the S group, while α‐linoleic acid metabolism and phenylpropanoid biosynthesis pathways were enhanced in the PR group (Figure 2C). Based on GO and KEGG pathway enrichment, it was apparent that the S group samples had a higher expression level of hypersensitive (HR) and program cell death (PCD) related genes. On the other hand, the expression of phenylpropanoid and terpene synthesis genes was enriched in the PR group.

### 3.3 Other Potential Resistant Factors for Hp Inhibition

**Figure 3:**
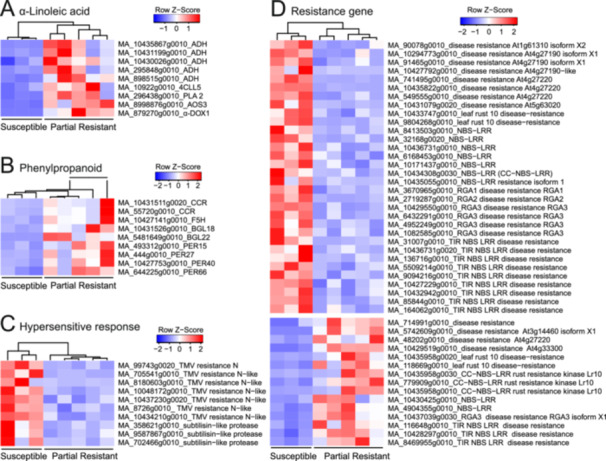
Heatmap of α‐linoleic acid metabolism, phenylpropanoid synthesis, hypersensitive response, and other resistance related genes that differential expressed. (A) α‐linoleic acid metabolism related genes that differentially expressed. (B) Phenylpropanoid synthesis related genes that differentially expressed. (C) Hypersensitive response genes that differentially expressed. The expression of transcripts that encoding for TMV resistance N (‐like) and subtilisin‐like protease were all upregulated in susceptible group. (D) Other resistance related genes that differentially expressed. The expression of transcripts that encoding for disease resistance proteins, including RGA disease resistance and NBS‐LRR disease resistance proteins. The expression of related genes and total read counts were taken into heatmap production with DESeq. 2 package. Raw count of transcripts with less than (<=) 5 in more than (>=) 80% samples were removed. Read counts were normalized and transformed with variance stabilizing transformation (vst) method. ADH, alcohol dehydrogenase; 4CLL5, 4‐coumarate ligase‐like 5; PLA2, phospholipase a2‐alpha; AOS3, allene oxide synthase 3‐like; α‐DOX1, alpha‐dioxygenase 1. CCR, cinnamoyl‐CoA reductase; F5H, ferulate 5‐hydroxylase; BGL, β−glucosidase; PER, peroxidase. [Color figure can be viewed at wileyonlinelibrary.com]

To depict the detailed transcriptomic differences, we investigated and grouped significant DEGs into certain categories, including HR and PCD related genes, resistance protein genes, terpene synthesis genes, phenylpropanoid synthesis genes, and so forth. The expression of α‐linoleic acid metabolism genes, which were suggested from KEGG analysis, were compared from S and PR groups. Nine transcripts (five genes) responsible for α‐linoleic acid metabolism were upregulated in the PR group (Figure 3A). In addition, we also observed that phenylpropanoid synthesis genes were generally highly expressed in the PR group (Figure 3B). On the other hand, significant differences in expression of genes related to HR and PCD between PR and S group. Genes related to HR that might contribute to necrosis, were highly expressed in S group (Figure 3C). Most strikingly, all the detected transcripts encoding for subtilisin‐like protease (3 transcripts) and TMV resistance N‐like proteins (seven transcripts) were upregulated in S group (Figure 3D). Moreover, many transcripts encoding for resistance proteins, NBS‐LRR resistance proteins, RGA (resistance gene analogs) resistance proteins were detected as differentially expressed genes (Figure 3D). Among these, more transcripts related to resistance genes were upregulated in S group (32 transcripts) than the PR group (15 transcripts) (Figure 3D).

### 3.4 Terpene Contents Were Distinct From Partial Resistant and Susceptible Clones

**Figure 4:**
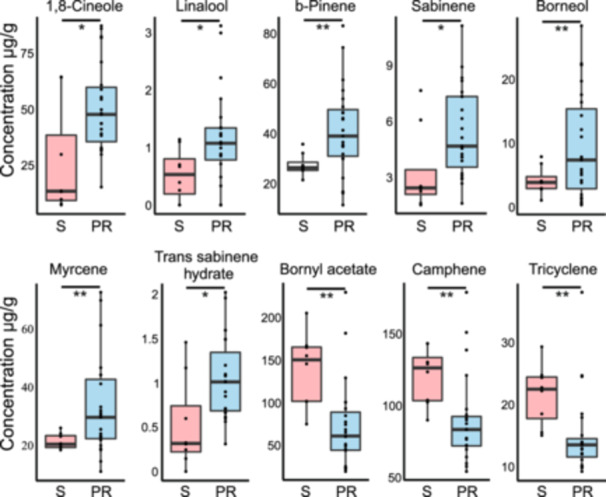
Terpene composition that significantly different in tolerant and susceptible groups. Among the tested monoterpenes and sesquiterpenes, the concentration of seven compounds were significantly higher in tolerant (T) group (*n* = 23) and three compounds were significantly higher in susceptible (S) group (*n* = 8). Comparison between two groups using terpene concentration of all clone individuals were conducted with two‐tailed unpaired *t*‐test in R, middle line represented the median, the box upper and lower ends were 75% and 25%, dots are outliers. PR, partial resistant group; S, susceptible group. *p < 0.05, **p < 0.01. [Color figure can be viewed at wileyonlinelibrary.com]

To correlate Norway spruce tolerance to their metabolites, we investigated the terpene production of the samples in PR and S groups. Average concentration of tested terpenes were compared. In general, more terpene compounds were significantly enriched in the PR group, including 1,8‐cineole, linalool, sabinene, trans sabinene hydrate, β‐pinene, myrcene, and borneol. Other terpene compounds including tricyclene, camphene and bornyl acetate were highly accumulated in S group (Figure 4).

**Figure 5:**
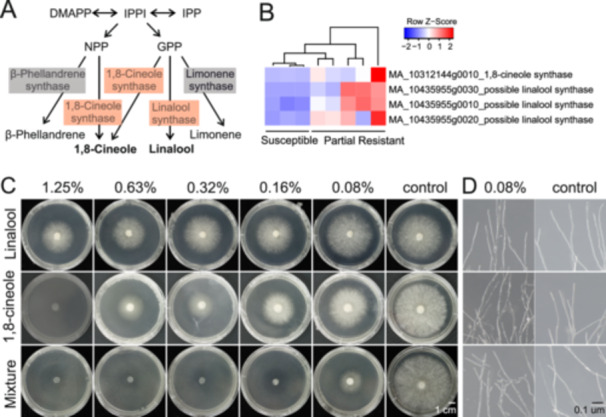
Linalool and 1,8‐cineole biosynthesis pathways and their inhibitory effect. (A) The biosynthesis pathways of four terpenes 1,8‐cineole, linalool, linalool and limonene in plants. The expression of possible linalool and 1,8‐cineole synthase genes were enriched in partial resistant group, which were highlighted in red. The genes of β‐phellandrene and limonene synthases were not detected as differential expressed genes. (B) Heatmap of differential expressed genes of possible linalool and 1,8‐cineole synthases in tolerant and susceptible groups. (C) Inhibitory effect of linalool, 1,8‐cineole and mixture against pathogen *H. parviporum*. (D) Disordered hyphae morphology of *H. parviporum* treated with linalool, 1,8‐cineole and mixture. DMAPP, dimethylallyl pyrophosphate; GPP, geranyl diphosphate; IPP, isopentenyl diphosphate; IPPI, isopentenyl diphosphate isomerase; MEP, methyl‐erythritol‐4‐phosphate; MVA, mevalonate; NPP, neryl diphosphate. [Color figure can be viewed at wileyonlinelibrary.com]

Notably, the average production of terpene linalool and 1,8‐cineole was significantly higher in the PR group than the S group. In agreement with that, the expression of 1,8‐cineole synthase gene and possible linalool synthase genes were also upregulated in the PR group (Figure 5A,B). The combination of chemical content and synthase gene expression of the two terpene compounds indicated that linalool and 1,8‐cineole were important chemical determinants of Norway spruce constitutive disease resistance factors.

### 3.5 Antifungal Effect of Terpenes Linalool and 1,8‐Cineole

To prove the potential role of terpenes linalool and 1,8‐cineole as resistance factors, we tested their inhibitory effect on Hp growth. Serial diluted concentrations of 1,8‐cineole, linalool and mixtures of the two terpene were set‐up to compare the growth inhibitory effect. Hp growth was totally blocked with 1.25% (v/v) of 1,8‐cineole and 0.16% (v/v) linalool and 1,8‐cineole mixture on PDA media (Figure 5C), indicating that the inhibitory effect with two terpene mixture was much stronger than a single terpene. The lowest linalool and 1,8‐cineole concentration for significant inhibition effect was 0.16% (v/v) (Figure 5C). Moreover, Hp hyphae was morphologically distorted when treated with 1,8‐cineole, linalool or two terpene mixtures, with atypical morphology such as hyphae curling, increased hyphal branching and deficient cell wall/membrane (Figure 5C).

### 3.6 Impact of Linalool and 1,8‐Cineole Treatment on Heterobasidion Gene Expression

To reveal how the spread of *Heterobasidion* could be inhibited in partial resistant clones, we performed RNAseq analysis from samples of Hp treated with linalool or linalool and 1,8‐cineole combined. Consistent with the inhibited phenotypes, linalool treated Hp showed significantly inhibited growth. Treatment of linalool and 1,8‐cineole mixture exhibited even stronger inhibition effect compared to control or linalool‐treated Hp.

**Figure 6:**
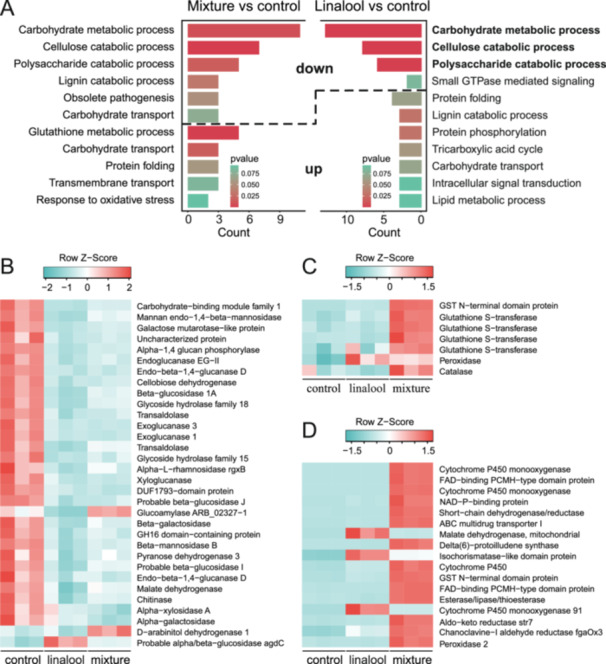
GO enrichment and differential expressed genes in terpene‐treated *Heterobasidion parviporum*. (A) GO term enrichment in mixture terpene‐treated and linalool‐treated *Heterobasidion parviporum* compared to control. (B–D) heatmap of differential expressed genes in mixture terpene‐treated and linalool‐treated *Heterobasidion parviporum* compared to control. Raw count of transcripts with less than (<=) 5 in more than (>=) 80% samples were removed. Read counts were normalized and transformed with variance stabilizing transformation (vst) method. Heatmap color was scaled by row. [Color figure can be viewed at wileyonlinelibrary.com]

Transcriptomic analysis revealed that the carbon assimilation (carbohydrate metabolic process, cellulose catabolic process and polysaccharide catabolic process) of Hp were largely inhibited by linalool and mixture treatment. Hp treated with two terpene mixture suffered more stress, indicated by the upregulation of genes in pathways of glutathione metabolic process and response to oxidative stress (Figure 6A,B). Significantly, multiple genes encoding for glutathione s‐transferase were uniquely upregulated in the chemical mixture than control or 1,8‐cineole treatment (Figure 6C). In addition, several cytochrome P450 monooxygenase and peroxidase genes were also upregulated in the mixture treatment (Figure 6D).

Furthermore, we investigated the effect of linalool and 1,8‐cineole on the expression of Hp effector genes. Notably, glycoside hydrolase genes were significantly downregulated at both linalool and combined treatment. However, some other effector genes were upregulated with terpenes treatment, such as thaumatin‐like protein and histidine kinase genes. These effector genes might be responsible for stress tolerance. Yet, many other effector genes with unknown functions were influenced by this two‐terpene treatment (Figure S4).

### 3.7 Gene Modules for the Tolerance Trait

**Figure 7:**
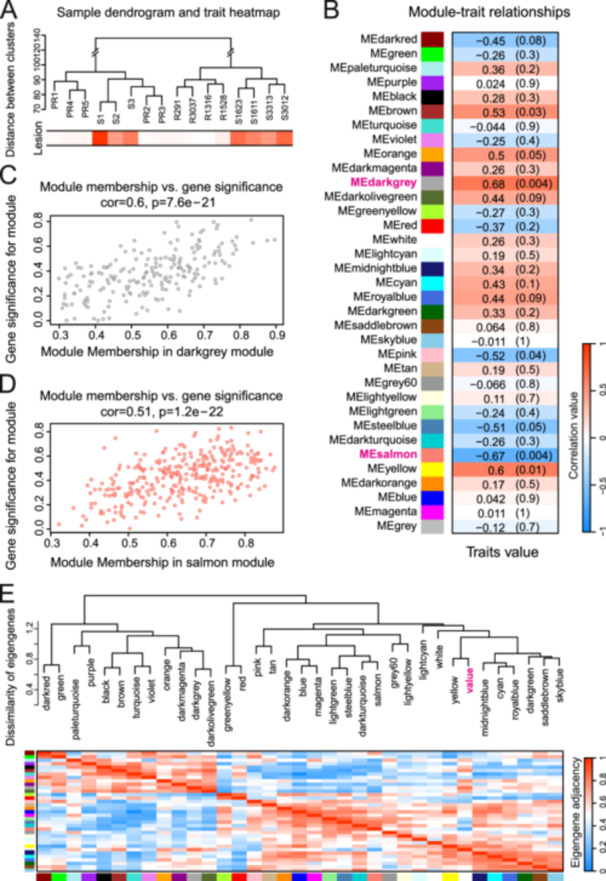
Weighted gene co‐expression network analysis (WGCNA) of transcriptome from tolerance and susceptible groups. (A) Sample dendrogram and trait heatmap based on lesion length. (B) Module‐trait relationships using gene modules and lesion length. Numbers represent the corresponding correlation and *p* value. (C, D) Scatterplot of gene significance for lesion in darkgrey and salmon modules, which were the two most significant modules. (E) Visualization of hierarchical clustering dendrogram of eigengenes and the eigengene adjacency for the relationships among modules and the phenotype lesion area (value). [Color figure can be viewed at wileyonlinelibrary.com]

To explore the co‐expression pattern of tolerance related genes, we performed WGCNA among PR and S groups. In addition to the transcriptomic analysis of the samples in this study, we also combined the RNAseq data from our previous resistance screening. GWCNA analysis of the PR group (nine clones) and S group (seven clones) was performed to explore potential transcriptomic networks of tolerance trait. A total of 21 850 transcripts post filtering were applied to construct networks for the key modules of highly correlated genes. Samples were clustered well for the resistance trait (Figure 7A). In total 35 modules were identified with the soft‐threshold power of 6 (Figure 7B). The darkgrey and salmon modules were most significant modules that correlated with the lesion trait, with coefficient values of 0.69 and −0.67. 199 hub genes in darkgrey module and 321 hub genes in salmon module (Figure 7C,D). The relationship of the tolerance traits and modules were present with a heatmap of hub gene adjacency (Figure 7E). Multiple gene modules showed significant correlation with Norway spruce, providing many more potential target genes that might contribute to resistance trait than we currently realized.

## 4 Discussion

### 4.1 Norway Spruce Resistance to Heterobasidion is a Quantitative Trait

We evaluated the potential resistance of Norway spruce against necrotrophic pathogen (*Heterobasidion* sp). Our screening showed the resistance phenotype (lesion length) was in a continuous mode, supporting the idea that tree resistance against necrotrophic pathogen is determined by multiple factors and complex networks. In addition, we investigated the correlation of lesion length and tree growth within the infection period. Nonsignificant correlation was detected between lesion development and tree height increase. This indicated that the resistance and growth trade‐off was not present at least within the infection period, which was consistent with our previous study (Liu et al. 2022).

Although resistance to *Heterobasidion* is a quantitative trait, we noticed that three S‐group clones had extremely large lesions (> 10‐fold in length) compared to other clones, as similar phenotype has been observed in our previous study (Kovalchuk et al. 2019). This suggests that susceptibility factors, like necrotic cell death might exist to benefit the growth and spread of Hp in sapwood and phloem. Comparison of transcriptomic and metabolic clues revealed potential susceptibility and resistance factors, which might determine the distinguished phenotypes of Norway spruce against necrotrophic pathogen Hp.

### 4.2 High Linalool and 1,8‐Cineole Content and Their Synthase Gene Expression in PR Group

Induced Norway spruce resistance by the infection of *Heterobasidion* is well studied (Kovalchuk et al. 2019; Chaudhary et al. 2020; Wen, Terhonen, and Asiegbu 2022). Yet, our knowledge of the constitutive resistance of Norway spruce is still rather limited. The chemical compositions of tree tissues are considered as a vital factor for tree resistance to pathogens and insects (Wang et al. 2023). Monoterpenes, including 1,8‐cineole and linalool, are present in multiple tissues of spruce and are internally transported via xylem sap (Duan, Bonn, and Kreuzwieser 2020). Monoterpene linalool, as a natural product from aromatic plants, has been widely studied in medical research for its bioactive properties, anti‐inflammatory, anticancer and antimicrobial functions (Pereira et al. 2018; Singh and Sharma 2015). As to the roles in plant biology, linalool is thought to have a major anti‐pathogen property. It has been shown to inhibit mycelial growth of a plant pathogen *Fusarium oxysporum*, with damage to pathogen cell membrane integrity and depression in its multiple metabolic pathways (Li et al. 2023). 1,8‐cineole has the inhibitory ability of biofilm and hyphae formation of *Fusarium solani* species complex (Zhang et al. 2022). Another study claimed that 1,8‐cineole act on organelles of *B. cinerea* and has synergistic effect with terpinen‐4‐ol for its anti‐fungal ability (Yu et al. 2015).

Our investigation of terpene composition of S and PR groups suggested that seven terpenes (1,8‐cineole, linalool, b‐pinene, sabinene, borneol, myrcene, and trans sabinene hydrate) were positively correlated and three terpenes (Bornyl acetate, camphene, and tricyclene) were negatively correlated with Hp partial resistance. Most significantly, we detected the higher expression level of 1,8‐cineole synthase gene and possible linalool synthase genes in the PR group. Therefore, we proposed that production and accumulation of linalool and 1,8‐cineole might contribute to Norway spruce partial resistance to necrotrophic pathogen Hp.

### 4.3 Antifungal Effect of Terpenes Linalool and 1,8‐Cineole

A few decades ago, antimicrobial activities of multiple monoterpenes were reported against fungal and/or bacterial strains (Mahizan et al. 2019; Dorman and Deans 2000; Kurita et al. 1979). Linalool and 1,8‐cineole were reported as multifunctional chemicals for plants (Li et al. 2023; Zhang et al. 2022; An et al. 2021). Volatile organic compounds have been found playing crucial roles in resisting biotic stresses of Norway spruce. For instance, Linalool is a monoterpene alcohol with antimicrobial and anti‐inflammatory properties, which is effective in inhibiting the growth of a wide range of pathogenic fungi and bacteria (Mączka et al. 2022). The synthesis of linalool is significantly induced and increased in Norway spruce when confronting biotic stress, thereby enhancing the plant's defenses (Blande, Turunen, and Holopainen 2009; Danielsson 2011). It has been found that linalool is able to interfere with the metabolic processes of pathogen, reducing their growth rate and thereby decreasing the damage to plant tissues (Li et al. 2023; Shimada et al. 2021). The 1,8‐cineole is another important monoterpene with strong antioxidant properties. This monoterpene can effectively resist a variety of necrotrophic pathogens, including the notorious Norway spruce pathogen *H. parviporum*. 1,8‐cineole protects trees by disrupting the pathogen's cell membranes and inhibiting its growth and reproduction (Kusumoto et al. 2014). From the perspective of insect resistance, Norway spruce secretes more 1,8‐cineole in the bark when attacked by European spruce bark beetle. This suggests that the potential of Norway spruce to induce defense compounds may partly determine its resistance to this pest, thus inhibiting the potential large‐scale attack (Schiebe et al. 2012). Previous studies have also suggested that linalool and 1,8‐cineole have positive effects on disease and pest resistance of Norway spruce. Yet, the antifungal effect of Linalool and 1,8‐cineole to the necrotroph Hp was not reported.

In this study, we observed direct inhibitory effect of terpenes linalool and 1,8‐cineole in vivo, which was consistent to the resistant phenotype of Norway spruce clones with higher linalool and 1,8‐cineole contents compared to susceptible clones. Here we propose that the synthesis of terpenes linalool and 1,8‐cineole could be constitutive resistance factors in Norway spruce against necrotrophic pathogen Hp, as it is evident from host transcriptomic and terpene metabolic levels, as well as pathogen direct inhibition and transcriptomic expression levels.

The normal growth of Hp was interrupted when treated with linalool or 1,8‐cineole, or mixtures of the two chemicals. The inhibitory concentration was a bit higher than those tested in our terpene analysis. However, considering the fact that local terpene concentration from the infection site can be higher than the average terpene concentration used in vitro, the concentration of linalool and 1,8‐cineole in Norway spruce tissues are efficient to limit the Hp invasion (Duan, Bonn, and Kreuzwieser 2020). The basal levels of linalool and 1,8‐cineole in the PR group were high enough to inhibit Hp growth.

Transcriptomic data suggests that potential pathogenicity factors of Hp was also negatively affected by linalool and 1,8‐cineole treatment. Glycoside hydrolases are pathogen effectors used to degrade plant tissues (Bradley et al. 2022). We discovered that effector genes encoding glycoside hydrolase were downregulated when treated with linalool and 1,8‐cineole. On the other hand, effector genes related to stress tolerance were upregulated with terpene treatment. Thaumatin‐like proteins are defense proteins in fungi (de Jesús‐Pires et al. 2020). Hp exhibited stress responses with upregulation of a thaumatin‐like protein gene when treated with terpenes, especially with linalool and 1,8‐cineole mixture. Yet, many other unknown effector genes were also influenced by terpene treatment. In this study, however, we did not include the transcriptomic analysis from 1,8‐cineole treated sample because of poor quality RNA harvested. This situation was probably raised from the inhibition of RNA synthesis or partial degradation of fungal RNA, or from the interference of the 1,8‐cineol terpene in the isolation of high‐quality RNA.

### 4.4 High Programmed Cell Death‐Related Gene Expression in S Group

Hypersensitive cell death (HR) and associated necrotic cell death was reported to facilitate the infection by necrotrophic pathogens *Botrytis cinerea* and *Sclerotinia sclerotiorum* (Govrin and Levine 2000). Similarly, the clones that exhibited high levels of necrotic related cell death, showed extremely low resistance against *H. parviporum* in this study. Some subtilisin‐like proteases have active role in plant PCD (Figueiredo, Sousa Silva, and Figueiredo 2018), which contribute to plant resistance against biotrophic pathogens. TMV resistance N‐like proteins are typical resistance proteins that elicit hypersensitive necrotic response in plants (Levy, Edelbaum, and Sela 2004). HR and PCD are effective approaches for plants to stop the establishment of infection by biotrophic pathogens. The higher expression of several transcripts encoding for subtilisin‐like proteases and TMV resistance N‐like proteins in S group indicated a high potential to initiate either a HR, PCD or necrotic cell death when infected with Hp. The initiation of necrotic cell death and associated HR and PCD, however, might benefit necrotrophic pathogen Hp to spread by utilizing dead plant tissues.

Similarly, more transcripts encoding for resistance proteins, NBS‐LRR resistance proteins, RGA resistance proteins were enriched in the S groups than the PR groups. These resistance proteins typically recognize pathogen effector proteins, which might lead to resistance responses including necrotic cell death, HR and PCD. However, this basic level gene expression is similar to anti‐biotrophic resistance genes that existed in some Norway spruce clones (S group) that correlated with Hp susceptibility. We showed that the expression of anti‐biotrophic resistance genes did not contribute to the tree partial resistance against necrotrophic pathogen.

### 4.5 Other Potential Resistant Factors for Hp Inhibition

Linoleic acid is the precursor of jasmonic acid, which is a signaling hormone that mediates plant resistance against necrotrophic pathogens. Constitutive higher expression of linoleic acid related genes in partial resistant group might represent a resistance factor. Phenylpropanoids are important metabolic compounds in plant resistance (Dong and Lin 2021; Kolosova and Bohlmann 2012; Shimada et al. 2021). We discovered that phenylpropanoid synthesis genes were upregulated in partial resistant group, indicating the constitutive resistant role of phenylpropanoids. Hypersensitive response was a mechanism for plant to quickly limit the pathogen spread (Heath 1998; Glazebrook 2005). However, hypersensitive response might facilitate the growth of necrotrophic pathogen. In this study, we found a significant higher expression pattern of hypersensitive response related genes of Norway spruce in Hp susceptible group. This finding supports the idea that hypersensitive response in trees was a susceptible factor for necrotrophic pathogen, which might lead to the extreme extensive lesion development.

We also investigated many other homologs of disease resistance genes and noticed that more disease resistance genes were highly expressed in the S group (Chang, Puryear, and Cairney 1993) than the PR group (Martin et al. 2002). Like HR genes, these genes are mostly responsible for resistance to biotrophic pathogen and play opposite function in trees when facing necrotrophic pathogen. Additionally, genes related to cell wall enrichment might also contribute to Hp resistance. For instance, β‐glucosidases are involved in formation of required intermediates for cell wall lignification and defense (Ketudat Cairns et al. 2015). Recent studies have suggested xyloglucan endotransglucosylase/hydrolase (XTH) are responsible for regulation of cell wall xyloglucan polymers and stress resistance (Ishida and Yokoyama 2022). Transcripts encoding for β‐glucosidases and XTH were upregulated in the PR group, indicating the probably active roles of these genes in Norway spruce partial resistance to Hp. In this study, the expression of flavonoid biosynthesis pathway was upregulated, while phenylpropanoid biosynthesis pathway was downregulated in the S group. These were contrary to our previous reports by Kovalchuk et al (Axelsson et al. 2020) and Liu et al (Kolosova and Bohlmann 2012), which was possibly because of the gene redundancy involving these pathways and the complexity of tree resistance to necrotrophic pathogen. The 3‐carene was proposed as a resistance factor in previous studies (Fäldt et al. 2003) and in Sitka spruce (Robert et al. 2010; Roach et al. 2014). However, the expression of (+)‐3‐carene synthase genes was not detected as DEG, suggesting that (+)‐3‐carene synthesis was not a constitutive resistance factor in Norway spruce.

### 4.6 Host Family on Hp Tolerance

Previous study indicated that host family influence the terpene profiles and fungal endophytes, with indirect impact on the disease resistance outcome (Bullington et al. 2018). In our study, the Norway spruce resistance to Hp from the view of clonal family also exhibited differential levels. Several families had significantly different resistance performance. However, huge variation of resistant levels is obvious in most of 17 clonal families, indicating that maternal parent identity has positive but limited role to Hp resistance performance. There was no correlation of tree growth and lesion length in this study. Similar results were found in our previous screening of 3‐year‐old Norway spruce (Liu et al. 2022). Taken together, these indicated that the lesion development does not affect the growth of young trees at least during the infection period in these young seedlings.

## 5 Conclusion

Although *Heterobasidion* is known as a root and stem rot pathogen, our previous study (Mukrimin et al. 2019) using Fourier‐transform infrared spectroscopy (FT‐IR) for analysis of 18 needle samples from asymptomatic and symptomatic Norway spruce trees, showed that FT‐IR spectra could be used to distinguish needle tissue extracts of symptomatic and asymptomatic *Heterobasidion* infected trees. Consequently, this observation strengthened the use of needles as a nondestructive sampling strategy. The result showed that some terpene content and their related gene expression were associated to Hp partial resistance phenotype, indicating the terpene (linalool and 1,8‐cineole) synthesis as possible constitutive characters of Hp resistance. Hypersensitive and necrotic responses are constitutive characteristic or pre‐infection features similar with other biotic factors. Hypersensitive and necrosis cell death was reported to facilitate the infection by necrotrophic pathogens *Botrytis cinerea* and *Sclerotinia sclerotiorum* (Li et al. 2023). Induction of necrotic cell death, hypersensitive response and programmed cell death in Norway spruce might facilitate necrotrophic pathogen Hp invasive growth in Norway spruce wood. Thus, we propose that the basal levels of terpenes (linalool and 1,8‐cineole) constitutive disease resistance factors for spruce against necrotrophic pathogen Hp. On the other hand, the basal expression level of necrosis related cell death genes are possible susceptible predisposing factors that lead to fast necrotic lesion development induced by the necrotrophic pathogen Hp.

## Conflicts of Interest

The authors declare no conflicts of interest.

## Supporting information

Supporting information.

Supporting information.

## Data Availability

The data that supports the findings of this study are available in the supplementary material of this article.

## References

[pce15280-bib-0001] An, Q. , J.‐N. Ren , X. Li , et al. 2021. “Recent Updates on Bioactive Properties of Linalool.” Food & Function 12, no. 21: 10370–10389. 34611674 10.1039/d1fo02120f

[pce15280-bib-0002] Anders, S. , P. T. Pyl , and W. Huber . 2015. “HTSeq‐A Python Framework to Work With High‐Throughput Sequencing Data.” Bioinformatics 31, no. 2: 166–169. 25260700 10.1093/bioinformatics/btu638 PMC4287950

[pce15280-bib-0003] Asiegbu, F. O. , A. Adomas , and J. Stenlid . 2005. “Conifer Root and Butt Rot Caused By *Heterobasidion annosum* (Fr.) Bref. sl.” Molecular Plant Pathology 6, no. 4: 395–409. 20565666 10.1111/j.1364-3703.2005.00295.x

[pce15280-bib-0004] Axelsson, K. , A. Zendegi‐Shiraz , G. Swedjemark , A.‐K. Borg‐Karlson , and T. Zhao . 2020. “Chemical Defence Responses of Norway Spruce to Two Fungal Pathogens.” Forest Pathology 50, no. 6: e12640.

[pce15280-bib-0005] Bates, D. , M. Mächler , B. Bolker , and S. Walker . 2015. “Fitting Linear Mixed‐Effects Models Using lme4.” Journal of Statistical Software 67, no. 1: 48.

[pce15280-bib-0006] Blande, J. D. , K. Turunen , and J. K. Holopainen . 2009. “Pine Weevil Feeding on Norway Spruce Bark Has a Stronger Impact on Needle VOC Emissions Than Enhanced Ultraviolet‐B Radiation.” Environmental Pollution 157, no. 1: 174–180. 18775595 10.1016/j.envpol.2008.07.007

[pce15280-bib-0007] Bolger, A. M. , M. Lohse , and B. Usadel . 2014. “Trimmomatic: A Flexible Trimmer for Illumina Sequence Data.” Bioinformatics 30, no. 15: 2114–2120. 24695404 10.1093/bioinformatics/btu170 PMC4103590

[pce15280-bib-0008] Bonello, P. , T. R. Gordon , D. A. Herms , D. L. Wood , and N. Erbilgin . 2006. “Nature and Ecological Implications of Pathogen‐Induced Systemic Resistance in Conifers: A Novel Hypothesis.” Physiological and Molecular Plant Pathology 68, no. 4–6: 95–104.

[pce15280-bib-0009] Bradley, E. L. , B. Ökmen , G. Doehlemann , B. Henrissat , R. E. Bradshaw , and C. H. Mesarich . 2022. “Secreted Glycoside Hydrolase Proteins as Effectors and Invasion Patterns of Plant‐Associated Fungi and Oomycetes.” Frontiers in Plant Science 13. 10.3389/fpls.2022.853106. PMC8960721 35360318

[pce15280-bib-0010] Bu, D. , H. Luo , P. Huo , et al. 2021. “KOBAS‐I: Intelligent Prioritization and Exploratory Visualization of Biological Functions for Gene Enrichment Analysis.” Nucleic Acids Research 49, no. W1: W317–W325. 34086934 10.1093/nar/gkab447 PMC8265193

[pce15280-bib-0011] Bullington, L. S. , Y. Lekberg , R. Sniezko , and B. Larkin . 2018. “The Influence of Genetics, Defensive Chemistry and the Fungal Microbiome on Disease Outcome in Whitebark Pine Trees.” Molecular Plant Pathology 19, no. 8: 1847–1858. 29388309 10.1111/mpp.12663 PMC6638087

[pce15280-bib-0012] Caudullo, G. , W. Tinner , and D. de Rigo . 2016. “ *Picea abies* in Europe: Distribution, Habitat, Usage and Threats.” In European Atlas of Forest Tree Species, edited by D. San‐Miguel‐Ayanz , G. Caudullo , T. Houston Durrant , and A. Mauri , 114–116. Luxembourg: Publication Office of the European Union.

[pce15280-bib-0013] Chang, S. , J. Puryear , and J. Cairney . 1993. “A Simple and Efficient Method for Isolating RNA From Pine Trees.” Plant Molecular Biology Reporter 11, no. 2: 113–116.

[pce15280-bib-0014] Chaudhary, R. , K. Lundén , K. Dalman , et al. 2020. “Combining Transcriptomics and Genetic Linkage Based Information to Identify Candidate Genes Associated With *Heterobasidion*‐Resistance in Norway Spruce.” Scientific Reports 10, no. 1: 12711. 32728135 10.1038/s41598-020-69386-0 PMC7391732

[pce15280-bib-0015] Chisholm, S. T. , G. Coaker , B. Day , and B. J. Staskawicz . 2006. “Host‐Microbe Interactions: Shaping the Evolution of the Plant Immune Response.” Cell 124, no. 4: 803–814. 16497589 10.1016/j.cell.2006.02.008

[pce15280-bib-0016] Dangl, J. L. , and J. D. G. Jones . 2001. “Plant Pathogens and Integrated Defence Responses to Infection.” Nature 411, no. 6839: 826–833. 11459065 10.1038/35081161

[pce15280-bib-0017] Danielsson, M. 2011. Chemical Defence in Norway spruce. Stockholm: Royal Institute of Technology.

[pce15280-bib-0018] Dobin, A. , C. A. Davis , F. Schlesinger , et al. 2013. “STAR: Ultrafast Universal RNA‐Seq Aligner.” Bioinformatics 29, no. 1: 15–21. 23104886 10.1093/bioinformatics/bts635 PMC3530905

[pce15280-bib-0019] Dong, N. Q. , and H. X. Lin . 2021. “Contribution of Phenylpropanoid Metabolism to Plant Development and Plant‐Environment Interactions.” Journal of Integrative Plant Biology 63, no. 1: 180–209. 33325112 10.1111/jipb.13054

[pce15280-bib-0020] Donoso, A. , V. Rodriguez , A. Carrasco , R. Ahumada , E. Sanfuentes , and S. Valenzuela . 2015. “Relative Expression of Seven Candidate Genes for Pathogen Resistance on *Pinus Radiata* Infected With *Fusarium Circinatum* .” Physiological and Molecular Plant Pathology 92: 42–50.

[pce15280-bib-0021] Dorman, H. J. D. , and S. G. Deans . 2000. “Antimicrobial Agents From Plants: Antibacterial Activity of Plant Volatile Oils.” Journal of Applied Microbiology 88, no. 2: 308–316. 10736000 10.1046/j.1365-2672.2000.00969.x

[pce15280-bib-0022] Duan, Q. , B. Bonn , and J. Kreuzwieser . 2020. “Terpenoids are Transported in the Xylem Sap of Norway Spruce.” Plant, Cell & Environment 43, no. 7: 1766–1778. 10.1111/pce.13763 32266975

[pce15280-bib-0023] Fäldt, J. , D. Martin , B. Miller , S. Rawat , and J. Bohlmann . 2003. “Traumatic Resin Defense in Norway Spruce (*Picea abies*): Methyl Jasmonate‐Induced Terpene Synthase Gene Expression, and Cdna Cloning and Functional Characterization of (+)‐3‐carene Synthase.” Plant Molecular Biology 51, no. 1: 119–133. 12602896 10.1023/a:1020714403780

[pce15280-bib-0024] Figueiredo, J. , M. Sousa Silva , and A. Figueiredo . 2018. “Subtilisin‐Like Proteases in Plant Defence: The Past, the Present and Beyond.” Molecular Plant Pathology 19, no. 4: 1017–1028. 28524452 10.1111/mpp.12567 PMC6638164

[pce15280-bib-0025] Garbelotto, M. , and P. Gonthier . 2013. “Biology, Epidemiology, and Control of Heterobasidion Species Worldwide.” Annual Review of Phytopathology 51: 39–59. 10.1146/annurev-phyto-082712-102225 23642002

[pce15280-bib-0026] Gilmour, A. , B. Gogel , B. Cullis , S. Welham , and R. Thompson . 2015. ASReml User Guide Release 4.1 Structural Specification. Hemel Hempstead: VSN International Ltd.

[pce15280-bib-0027] Glazebrook, J. 2005. “Contrasting Mechanisms of Defense Against Biotrophic and Necrotrophic Pathogens.” Annual Review of Phytopathology 43, no. 1: 205–227. 10.1146/annurev.phyto.43.040204.135923 16078883

[pce15280-bib-0028] Govrin, E. M. , and A. Levine . 2000. “The Hypersensitive Response Facilitates Plant Infection by the Necrotrophic Pathogen *Botrytis Cinerea* .” Current Biology 10, no. 13: 751–757. 10898976 10.1016/s0960-9822(00)00560-1

[pce15280-bib-0029] Heath, M. C. 1998. “Apoptosis, Programmed Cell Death and the Hypersensitive Response.” European Journal of Plant Pathology 104, no. 2: 117–124.

[pce15280-bib-0030] Ishida, K. , and R. Yokoyama . 2022. “Reconsidering the Function of the Xyloglucan Endotransglucosylase/Hydrolase Family.” Journal of Plant Research 135, no. 2: 145–156. 35000024 10.1007/s10265-021-01361-w

[pce15280-bib-0031] Isik, F. , J. Holland , and C. Maltecca . 2017. Genetic Data Analysis for Plant and Animal breeding. Cham, Switzerland: Springer International Publishing.

[pce15280-bib-0032] de Jesús‐Pires, C. , J. R. C. Ferreira‐Neto , J. Pacifico Bezerra‐Neto , et al. 2020. “Plant Thaumatin‐Like Proteins: Function, Evolution and Biotechnological Applications.” Current Protein & Peptide Science 21, no. 1: 36–51. 30887921 10.2174/1389203720666190318164905

[pce15280-bib-0033] Kainulainen, P. , J. Oksanen , V. Palomäki , J. K. Holopainen , and T. Holopainen . 1992. “Effect of Drought and Waterlogging Stress on Needle Monoterpenes of *Picea Abies* .” Canadian Journal of Botany 70, no. 8: 1613–1616.

[pce15280-bib-0034] Kärhä, K. , V. Koivusalo , T. Palander , and M. Ronkanen . 2018. “Treatment of *Picea abies* and *Pinus Sylvestris* Stumps With Urea and *Phlebiopsis Gigantea* for Control of *Heterobasidion* .” Forests 9, no. 3: 139.

[pce15280-bib-0035] Keeling, C. I. , and J. Bohlmann . 2006. “Genes, Enzymes and Chemicals of Terpenoid Diversity in the Constitutive and Induced Defence of Conifers Against Insects and Pathogens.” New Phytologist 170, no. 4: 657–675. 16684230 10.1111/j.1469-8137.2006.01716.x

[pce15280-bib-0036] Ketudat Cairns, J. R. , B. Mahong , S. Baiya , and J. S. Jeon . 2015. “β‐Glucosidases: Multitasking, Moonlighting or Simply Misunderstood?” Plant Science 241: 246–259. 26706075 10.1016/j.plantsci.2015.10.014

[pce15280-bib-0037] Kolosova, N. , and J. Bohlmann . 2012. “Conifer Defense Against Insects and Fungal Pathogens.” In Growth and Defence in Plants: Resource Allocation at Multiple Scales, edited by R. Matyssek , H. Schnyder , W. Oßwald , D. Ernst , J. C. Munch , and H. Pretzsch , 85–109. Berlin, Heidelberg: Springer Berlin Heidelberg.

[pce15280-bib-0038] Kopylova, E. , L. Noé , and H. Touzet . 2012. “Sortmerna: Fast and Accurate Filtering of Ribosomal RNAs in Metatranscriptomic Data.” Bioinformatics 28, no. 24: 3211–3217. 23071270 10.1093/bioinformatics/bts611

[pce15280-bib-0039] Kovalchuk, A. , S. Keriö , A. O. Oghenekaro , E. Jaber , T. Raffaello , and F. O. Asiegbu . 2013. “Antimicrobial Defenses and Resistance in Forest Trees: Challenges and Perspectives in a Genomic Era.” Annual Review of Phytopathology 51: 221–244. 10.1146/annurev-phyto-082712-102307 23682916

[pce15280-bib-0040] Kovalchuk, A. , Z. Zeng , R. P. Ghimire , et al. 2019. “Dual RNA‐Seq Analysis Provides New Insights Into Interactions Between Norway Spruce and Necrotrophic Pathogen *Heterobasidion Annosum S.L* .” BMC Plant Biology 19, no. 1: 2. 30606115 10.1186/s12870-018-1602-0 PMC6318961

[pce15280-bib-0041] Kurita, N. , M. Miyaji , R. Kurane , Y. Takahara , and K. Ichimura . 1979. “Antifungal Activity and Molecular Orbital Energies of Aldehyde Compounds From Oils of Higher Plants.” Agricultural and Biological Chemistry 43, no. 11: 2365–2371.

[pce15280-bib-0042] Kusumoto, N. , T. Zhao , G. Swedjemark , T. Ashitani , K. Takahashi , and A. K. Borg‐Karlson . 2014. “Antifungal Properties of Terpenoids in *Picea abies* against *Heterobasidion Parviporum* .” Forest Pathology 44, no. 5: 353–361.

[pce15280-bib-0043] Langfelder, P. , and S. Horvath . 2008. “WGCNA: An R Package for Weighted Correlation Network Analysis.” BMC Bioinformatics 9: 559. 19114008 10.1186/1471-2105-9-559 PMC2631488

[pce15280-bib-0044] Levy, M. , O. Edelbaum , and I. Sela . 2004. “Tobacco Mosaic Virus Regulates the Expression of its Own Resistance Gene N.” Plant Physiology 135, no. 4: 2392–2397. 15299123 10.1104/pp.104.044859 PMC520806

[pce15280-bib-0045] Li, X. , Q. Wang , H. Li , et al. 2023. “Revealing the Mechanisms for Linalool Antifungal Activity Against *Fusarium Oxysporum* and its Efficient Control of Fusarium Wilt in Tomato Plants.” International Journal of Molecular Sciences 24, no. 1: 458. 10.3390/ijms24010458 PMC9820380 36613902

[pce15280-bib-0046] Liu, M. , E. Jaber , Z. Zeng , A. Kovalchuk , and F. O. Asiegbu . 2021. “Physiochemical and Molecular Features of the Necrotic Lesion in Theheterobasidion–Norway Spruce Pathosystem.” Tree Physiology 41: 791–800. 33105481 10.1093/treephys/tpaa141

[pce15280-bib-0047] Liu, M. , K. Wang , R. P. Ghimire , M. Haapanen , M. Kivimäenpää , and F. O. Asiegbu . 2022. “Molecular and Chemical Screening for Inherent Disease Resistance Factors of Norway Spruce (*Picea abies*) Clones Against Conifer Stem Rot Pathogen Heterobasidion Parviporum.” Phytopathology 112, no. 4: 872–880. 34698543 10.1094/PHYTO-09-21-0379-R

[pce15280-bib-0048] Love, M. I. , W. Huber , and S. Anders . 2014. “Moderated Estimation of Fold Change and Dispersion for RNA‐Seq Data With Deseq. 2.” Genome Biology 15, no. 12: 550. 25516281 10.1186/s13059-014-0550-8 PMC4302049

[pce15280-bib-0049] Mageroy, M. H. , E. Christiansen , B. Långström , et al. 2020. “Priming of Inducible Defenses Protects Norway Spruce against Tree‐Killing Bark Beetles.” Plant, Cell & Environment 43, no. 2: 420–430. 10.1111/pce.13661 31677172

[pce15280-bib-0050] Mahizan, N. A. , S. K. Yang , C. L. Moo , et al. 2019. “Terpene Derivatives as a Potential Agent Against Antimicrobial Resistance (AMR) Pathogens.” Molecules 24, no. 14: 2631. 31330955 10.3390/molecules24142631 PMC6680751

[pce15280-bib-0051] Martin, D. , D. Tholl , J. Gershenzon , and J. Bohlmann . 2002. “Methyl Jasmonate Induces Traumatic Resin Ducts, Terpenoid Resin Biosynthesis, and Terpenoid Accumulation in Developing Xylem of Norway Spruce Stems.” Plant Physiology 129, no. 3: 1003–1018. 12114556 10.1104/pp.011001 PMC166496

[pce15280-bib-0052] Mukrimin, M. , A. O. Conrad , A. Kovalchuk , R. Julkunen‐Tiitto , P. Bonello , and F. O. Asiegbu . 2019. “Fourier‐Transform Infrared (FT‐IR) Spectroscopy Analysis Discriminates Asymptomatic and Symptomatic Norway Spruce Trees.” Plant Science 289: 110247. 31623795 10.1016/j.plantsci.2019.110247

[pce15280-bib-0053] Mączka, W. , A. Duda‐Madej , M. Grabarczyk , and K. Wińska . 2022. “Natural Compounds in the Battle against Microorganisms—Linalool.” Molecules 27, no. 20: 6928. 36296521 10.3390/molecules27206928 PMC9609897

[pce15280-bib-0054] Naidoo, S. , B. Slippers , J. M. Plett , D. Coles , and C. N. Oates . 2019. “The Road to Resistance in Forest Trees.” Frontiers in Plant Science 10. 10.3389/fpls.2019.00273. PMC6455082 31001287

[pce15280-bib-0055] Nemesio‐Gorriz, M. , A. Hammerbacher , K. Ihrmark , et al. 2016. “Different Alleles of a Gene Encoding Leucoanthocyanidin Reductase (*Palar3*) Influence Resistance Against the Fungus *Heterobasidion Parviporum* in *Picea Abies* .” Plant Physiol 171, no. 4: 2671–2681. 27317690 10.1104/pp.16.00685 PMC4972290

[pce15280-bib-0056] Novak, M. , A. U. Krajnc , L. Lah , et al. 2014. “Low‐Density *Ceratocystis Polonica* Inoculation of Norway Spruce (*Picea Abies*) Triggers Accumulation of Monoterpenes With Antifungal Properties.” European Journal of Forest Research 133, no. 4: 573–583.

[pce15280-bib-0057] Nystedt, B. , N. R. Street , A. Wetterbom , et al. 2013. “The Norway Spruce Genome Sequence and Conifer Genome Evolution.” Nature 497, no. 7451: 579–584. 23698360 10.1038/nature12211

[pce15280-bib-0058] Pereira, I. , P. Severino , A. C. Santos , A. M. Silva , and E. B. Souto . 2018. “Linalool Bioactive Properties and Potential Applicability in Drug Delivery Systems.” Colloids and Surfaces B: Biointerfaces 171: 566–578. 30098535 10.1016/j.colsurfb.2018.08.001

[pce15280-bib-0059] Roach, C. R. , D. E. Hall , P. Zerbe , and J. Bohlmann . 2014. “Plasticity and Evolution of (+)‐3‐carene Synthase and (‐)‐Sabinene Synthase Functions of a Sitka Spruce Monoterpene Synthase Gene Family Associated With Weevil Resistance.” Journal of Biological Chemistry 289, no. 34: 23859–23869. 25016016 10.1074/jbc.M114.571703 PMC4156071

[pce15280-bib-0060] Robert, J. A. , L. L. Madilao , R. White , A. Yanchuk , J. King , and J. Bohlmann . 2010. “Terpenoid Metabolite Profiling in Sitka Spruce Identifies Association of Dehydroabietic Acid, (+)‐3‐carene, and Terpinolene With Resistance against White Pine Weevil.” Botany 88, no. 9: 810–820.

[pce15280-bib-0061] Schiebe, C. , A. Hammerbacher , G. Birgersson , et al. 2012. “Inducibility of Chemical Defenses in Norway Spruce Bark is Correlated With Unsuccessful Mass Attacks by the Spruce Bark Beetle.” Oecologia 170, no. 1: 183–198. 22422313 10.1007/s00442-012-2298-8

[pce15280-bib-0062] Schneider, C. A. , W. S. Rasband , and K. W. Eliceiri . 2012. “NIH Image to Imagej: 25 Years of Image Analysis.” Nature Methods 9, no. 7: 671–675. 22930834 10.1038/nmeth.2089 PMC5554542

[pce15280-bib-0063] Shimada, T. , T. Endo , H. Fujii , et al. 2021. “Biological and Molecular Characterization of Linalool‐Mediated Field Resistance Against *Xanthomonas Citri* Subsp. Citri in Citrus Trees.” Tree Physiology 41: 2171–2188. 33960371 10.1093/treephys/tpab063

[pce15280-bib-0064] Singh, B. , and R. A. Sharma . 2015. “Plant Terpenes: Defense Responses, Phylogenetic Analysis, Regulation and Clinical Applications.” 3 Biotech 5, no. 2: 129–151. 10.1007/s13205-014-0220-2 PMC4362742 28324581

[pce15280-bib-0065] Sniezko, R. , J. Smith , J.‐J. Liu , and R. Hamelin . 2014. “Genetic Resistance to Fusiform Rust in Southern Pines and White Pine Blister Rust in White Pines—A Contrasting Tale of Two Rust Pathosystems—Current Status and Future Prospects.” Forests 5, no. 9: 2050–2083.

[pce15280-bib-0066] Sniezko, R. A. , and J. Koch . 2017. “Breeding Trees Resistant to Insects and Diseases: Putting Theory Into Application.” Biological Invasions 19, no. 11: 3377–3400.

[pce15280-bib-0067] Wang, K. , M. Liu , F. Cui , and F. Asiegbu . 2021. “A Simple Phenol‐Free Isolation Method for High‐Quality Rna From Bilberry.” MethodsX 8: 101481. 34434879 10.1016/j.mex.2021.101481 PMC8374723

[pce15280-bib-0068] Wang, K. , I. Miettinen , E. H. Jaber , and F. O. Asiegbu . 2023. “Chapter 2 ‐ Anatomical, Chemical, Molecular, and Genetic Basis for Tree Defenses.” In Forest Microbiology, edited by F. O. Asiegbu , and A. Kovalchuk , 33–57). 3: Academic Press.

[pce15280-bib-0069] Wang, K. , Z. Wen , and F. O. Asiegbu . 2022. “The Dark Septate Endophyte *Phialocephala Sphaeroides* Suppresses Conifer Pathogen Transcripts and Promotes Root Growth of Norway Spruce.” Tree Physiology 42, no. 12: 2627–2639. 35878416 10.1093/treephys/tpac089 PMC9743008

[pce15280-bib-0070] Warnes, G. , B. Bolker , L. Bonebakker , et al. 2024. *gplots: Various R Programming Tools for Plotting Data*. R package version 3.2.0. https://talgalili.github.io/gplots/.

[pce15280-bib-0071] Wen, Z. , E. Terhonen , and F. O. Asiegbu . 2022. “The Dark Septate Endophyte *Phialocephala Sphaeroides* Confers Growth Fitness Benefits and Mitigates Pathogenic Effects of *Heterobasidion* on Norway Spruce.” Tree Physiology 42, no. 4: 891–906. 34791486 10.1093/treephys/tpab147 PMC9000907

[pce15280-bib-0072] Wickham, H. 2011. “The Split‐Apply‐Combine Strategy for Data Analysis.” Journal of Statistical Software 40, no. 1: 1–29.

[pce15280-bib-0073] Woodcock, P. , M. Marzano , and C. P. Quine . 2019. “Key Lessons From Resistant Tree Breeding Programmes in the Northern Hemisphere.” Annals of Forest Science 76, no. 2: 51.

[pce15280-bib-0074] Wu, T. , E. Hu , S. Xu , et al. 2021. “Clusterprofiler 4.0: A Universal Enrichment Tool for Interpreting Omics Data.” The Innovation 2, no. 3: 100141. 34557778 10.1016/j.xinn.2021.100141 PMC8454663

[pce15280-bib-0075] Yu, D. , J. Wang , X. Shao , F. Xu , and H. Wang . 2015. “Antifungal Modes of Action of Tea Tree Oil and Its Two Characteristic Components Against *Botrytis Cinerea* .” Journal of Applied Microbiology 119, no. 5: 1253–1262. 26294100 10.1111/jam.12939

[pce15280-bib-0076] Zhang, Y. , Y. Wang , X. Zhao , et al. 2022. “Study on the Anti‐Biofilm Mechanism of 1,8‐cineole Against *Fusarium Solani* Species Complex.” Frontiers in Pharmacology 13: 1010593. 36330094 10.3389/fphar.2022.1010593 PMC9624185

