# Supplementary material for: Linalool and 1,8‐Cineole as Constitutive Disease‐Resistant Factors of Norway Spruce Against Necrotrophic Pathogen Heterobasidion Parviporum
Source: Plant Cell Environ. 2024 Nov 13;48(3):1993–2008. doi: 10.1111/pce.15280 (PMC11788960; doi:10.1111/pce.15280)
Supplement: Supplementary file 1 — Supporting information. [file PCE-48-1993-s002.pdf]

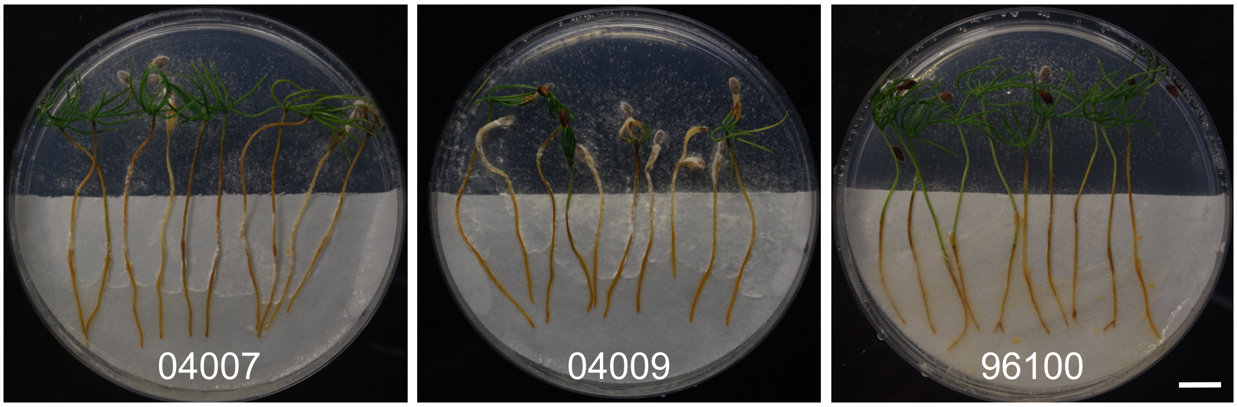

Supplementary figure 1. *Heterobasidion parviporum* virulence test of heterokaryotic isolates 04007, 04009 and 96100. Photos were taken 25 days post infection. Bar = 1 cm.

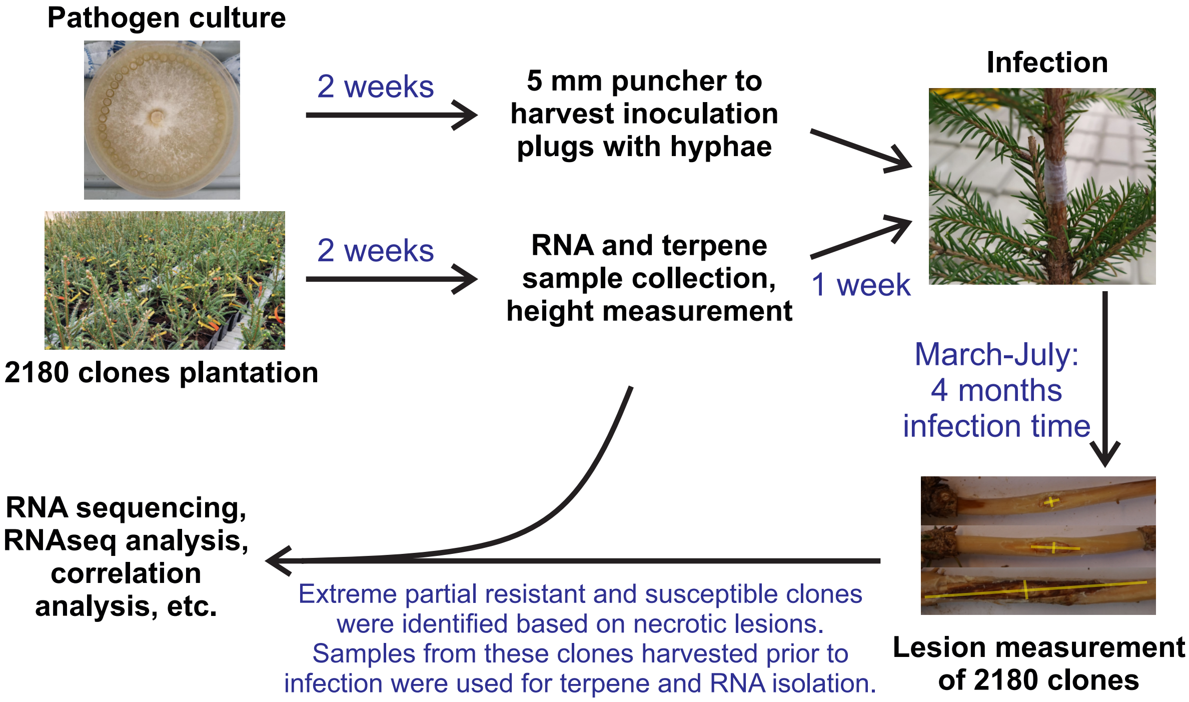

Supplementary figure 2. Experimental flow and time line in this study.

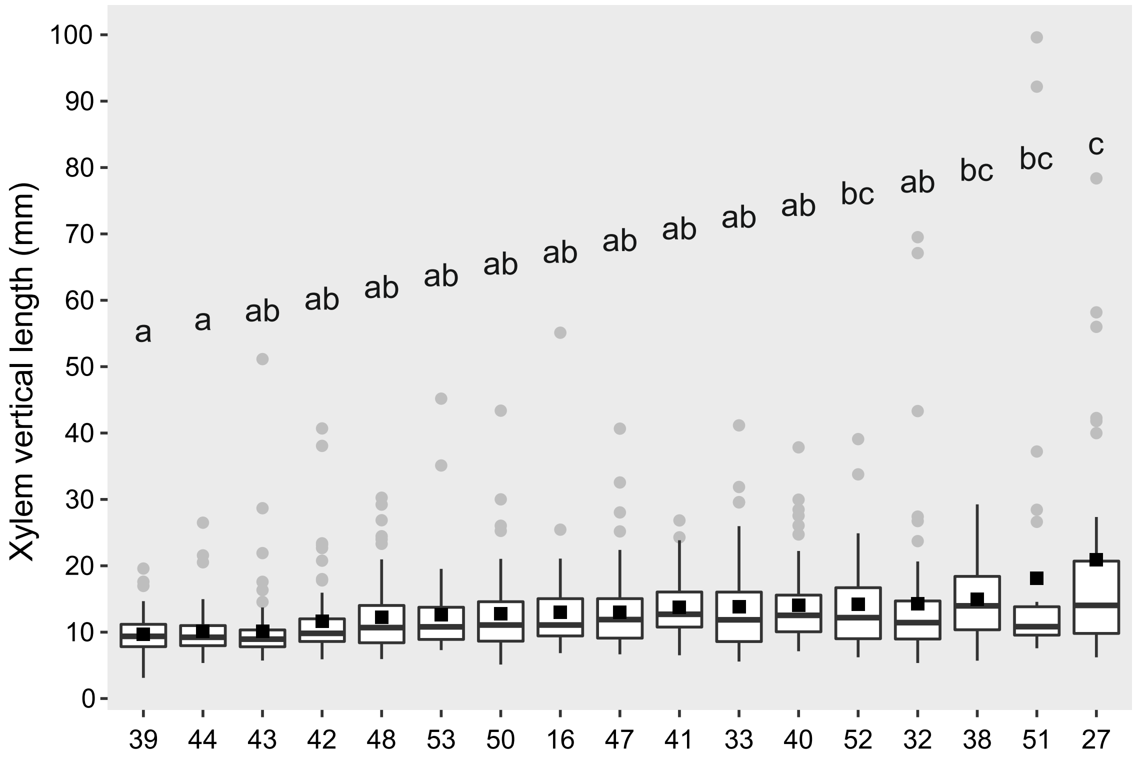

Supplementary figure 3. Summary of lesion xylem vertical length based on families. Lesion data was plotted with ggplot2 in R, middle line represented the median and black squarer indicated the means, the box upper and lower ends were 75% and 25% percentiles, grey dots mean the outliers. One-way ANOVA and Turkey HSD were conducted to test the differences among families, different letters indicated the significant different between samples.

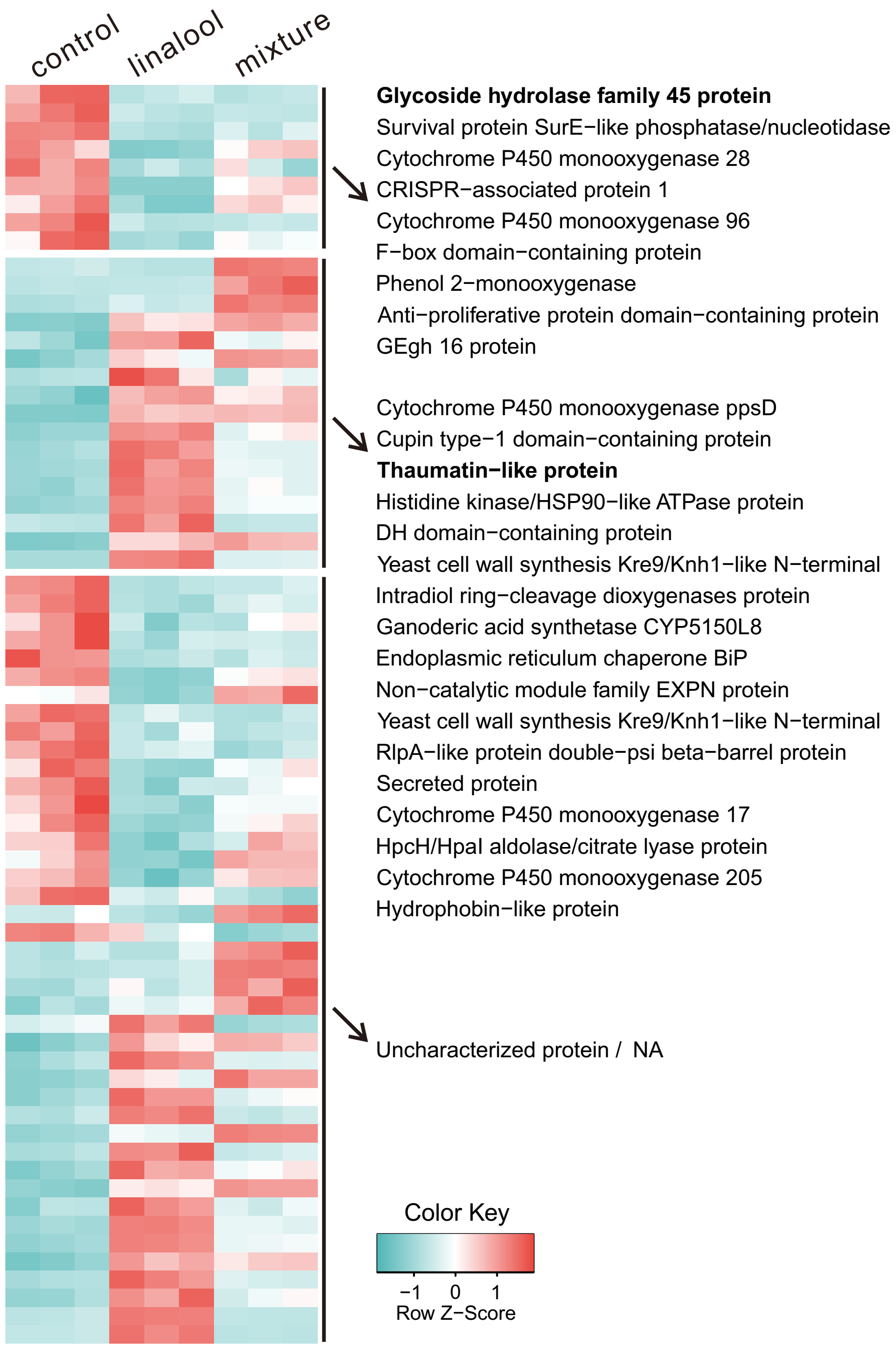

Supplementary figure 4. Heatmap of the expression of candidate effector genes in terpene-treated *Heterobasidion parviporum*. Raw count of transcripts with less than (<=) 5 in more than (>=) 80% samples were removed. Read counts were normalized and transformed with variance stabilizing transformation (vst) method. Heatmap colour was scaled by row.
